# Performance Evolution of CFRP Strip Anodes in Concrete: An Integrated Electrochemical and Mechanical Study

**DOI:** 10.3390/polym17182494

**Published:** 2025-09-16

**Authors:** Xuan Wu, Yichen Jia, Yingwu Zhou, Chengcheng Xue, Biao Hu, Yinghou He, Xiaoxu Huang

**Affiliations:** 1 School of Civil and Transportation Engineering, Shenzhen University, Shenzhen 518060, China; xuanw0805@163.com (X.W.); 13510484239@163.com (Y.J.); ywzhou@szu.edu.cn (Y.Z.); cexuecc@szu.edu.cn (C.X.); biaohu3-c@szu.edu.cn (B.H.); 2 Research Center for Wind Engineering and Engineering Vibration, Guangzhou University, Guangzhou 510006, China; heyinghou@gzhu.edu.cn

**Keywords:** impressed current cathodic protection, carbon fiber reinforced polymer, anodic polarization, electrochemical property, mechanical property

## Abstract

Impressed current cathodic protection (ICCP) is one of the most effective techniques in preventing steel corrosion in concrete structures. Based on the exceptional electrical conductivity and mechanical properties of carbon fiber reinforced polymers (CFRP), a novel structural system employing ICCP is proposed in this paper, in which CFRP strips are used as both concrete stirrups and as an auxiliary anode for cathodic protection. To further verify the dual functions of CFRP strips for this new system, the electrochemical and mechanical behaviors of the CFRP strip anode are investigated experimentally in this study through the anodic polarization test, electrochemical impedance spectroscopy test, uniaxial tensile test, and interfacial acidification test. The effects of concrete type and anode current density on the properties of CFRP strip anodes are identified. The results show that the CFRP strip anode possesses satisfactory electrical conductivity and relatively low output resistance, and the ultimate strength of the CFRP strip after polarization is reduced as the current density increases due to the gradual degradation of the CFRP anode. The mechanical properties of CFRP strips in Engineered Cementitious Cement (ECC) concrete and geopolymer concrete outperform those of ordinary concrete, and the degradation rate of CFRP strips subjected to anodic polarization in ECC concrete is lower than that of geopolymer concrete. The cathodic protection mechanism of CFRP strips as an anode is further revealed via numerical analysis. In addition, the prediction model of the service life is constructed for the proposed novel concrete structural system. The predicted service life of the system decreases as the reinforcement ratio increases, and it increases as the stirrup ratio increases. The predicted service life of the ICCP system in ECC concrete is significantly longer than that in geopolymer concrete and ordinary concrete.

## 1. Introduction

Corrosion is considered a key factor leading to the lack of durability for coastal concrete infrastructures [1]. Once the corrosive medium penetrates the concrete and destroys the passivation film formed on the surface of rebars, the corrosion process is accelerated. Steel corrosion is an electrochemical process that involves the dissolution of iron at the anode and the reduction of oxygen at the cathode [2]. Corrosion products generate expansion stresses that promote crack propagation. With the development of corrosion, the bearing capacity of reinforced concrete (RC) structures gradually weakens, resulting in a reduction in structural safety during service and even significant economic losses [3]. The global annual loss due to steel corrosion is reportedly up to USD 2.5 trillion, which accounts for about 3.4% of the world’s GDP. In China, the direct loss induced by road and bridge corrosion is about RMB 62.37 billion, representing 4.03% of the total investment in the entire industry [4]. Indeed, the maintenance costs of RC structures caused by corrosion cannot be ignored [5]. For example, the total number of bridges in need of repair in the Netherlands will be two to four times higher in 2042 than in 2020, placing a heavy burden on the economy [6].

Based on the corrosion mechanism, a variety of measures have been proposed to control steel corrosion [7], including the utilization of corrosion-resistant reinforcement (e.g., stainless steel reinforcement and fiber reinforced polymer [FRP] reinforcement), coated reinforcement, rust inhibitors, and electrochemical protection technologies (e.g., cathodic protection, electrochemical desalination). Among them, impressed current cathodic protection (ICCP) is an efficient measure that has been proven to completely inhibit steel corrosion in salt-contaminated concrete [8], and has been widely applied in major projects to ensure the long-term and safe service of structures. By forcing steel bars to become cathodes, sufficient electrons are transferred to the rebars’ surface with the corresponding potentials decreasing to more negative values, and steel corrosion is ultimately inhibited [9]. In addition, developing suitable auxiliary anode materials is important to the application of ICCP technology for RC structures in practical engineering.

Featuring light-weight, high-strength, corrosion-resistant, and conductive [10,11], carbon fiber reinforced polymer (CFRP) may be a suitable type of auxiliary anode material for ICCP systems in terms of CFRP grids, CFRP sheets, and CFRP bars. In recent years, the effectiveness of an CFRP anode in ICCP systems has been verified in material, interface, and structural aspects [12,13]. Lee-Orantes et al. [14] conducted the first experimental study on the cathodic protection of RC structures with CFRP as an anode, and the results showed that, as a type of external structural reinforcement, CFRP strips also exhibit effective cathodic protection simultaneously. Nguyen et al. [15] focused on the properties of CFRP anode materials (CFRP sheets and CFRP bars) in concrete and Ca(OH)2 solutions and found that carbon fiber is subject to a certain level of degradation under cathodic protection, and the degradation degree of CFRP sheets is higher than that of CFRP bars. Sun et al. [16] found that when focused CFRP sheets are in a NaCl solution, the epoxy resin decomposed due to the breakage of the C-N bond in the process of anodic polarization, which in turn caused the performance degradation of CFRP sheets. Zhu et al. [17] confirmed the feasibility of CFRP grids as auxiliary anode materials in the ICCP system based on a series of experiments. Zhou et al. [18] pointed out that CFRP anodes have a satisfactory service life in ICCP systems, with only 12% of strength loss after 62 years. Zhang et al. [19] conducted a study on the long-term degradation of the CFRP grid-concrete interface, and a nonlinear interface damage model for predicting the service life of the ICCP system was established. Lu et al. [20] proved that ICCP efficiency depends on the rebars’ pre-corrosion ratio. For RC columns wrapped with CFRP sheets, Gadve et al. [21,22] found that the steel–concrete bond performance decreased due to the damage of the rebars’ helical ribs.

In the last decade, the performance of CFRP anode materials in RC structures has been identified in ICCP and structural strengthening (i.e., dual functions). Lambert et al. [10] confirmed the dual functions of CFRP sheets for RC beams and pointed out that the U-shaped configuration can effectively reduce the debonding probability of the CFRP sheet anode. Zhu et al. [23] proposed an ICCP-structural strengthening (i.e., ICCP-SS) technology based on the CFRP grid. Aimed at corroded RC continuous beams, Su et al. [24] further proved the dual functions of ICCP-SS experimentally. Additionally, Feng et al. [25] and Wei et al. [26] experimentally investigated the fatigue performance of corroded RC beams treated with the ICCP-SS technique. Aimed at achieving safe and long-term service for existing marine structures, it is urgent to avoid steel corrosion and enhance structural performance, and the dual-function of CFRP material can be employed, such as CFRP-confined concrete columns [20,23]. Recently, the authors’ research group has proposed an ICCP-composite structural (i.e., ICCP-CS) system based on hybrid CFRP/steel reinforcements. Among them, CFRP bars are used not only as concrete reinforcing materials but also as anode materials to prevent steel corrosion. Compared with unprotected RC beams, the ultimate bending capacity increased by 25.1% for this system. Therefore, using CFRP materials as both anodes and structural reinforcement materials is feasible and effectively improves the durability of RC structures. The integrated structure-function ICCP-CS system has a promising application prospect and provides a brand-new technology for the development of high-durability marine engineering concrete structures.

**Figure 1:**
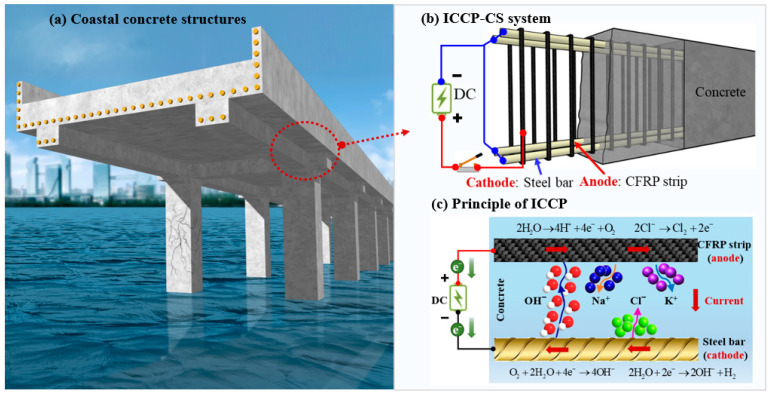
A novel RC structure with CFRP strips as stirrups and anodes.

To further promote the ICCP-CS system, a novel RC structure with a CFRP strip as a stirrup and anode is proposed in this study, as shown in Figure 1. The advantages of this system are multi-fold. (1) The dual functions (i.e., structural reinforcement and cathodic protection) of the CFRP strip can be fully demonstrated, which enhances the engineering application value of CFRP materials. (2) By replacing steel stirrups in RC structures with CFRP strips, the insufficient ductility and bond performance of the CFRP strip can be avoided. (3) The CFRP strip anode is embedded in the concrete without any adhesive, ensuring the exceptional bond performance of the anode–concrete interface.

In the following study, the electrochemical and mechanical properties of the CFRP strip anode were investigated experimentally and analytically. A series of experiments was conducted, including the constant current polarization test, the electrochemical impedance spectroscopy test, the uniaxial tensile test, and the interfacial acidification test. The effects of concrete type and anode current density on the properties of CFRP strip anodes were identified. To further reveal the cathodic protection mechanism of CFRP strips as an anode, numerical simulation analysis was performed using COMSOL 6.3 Multiphysics software. In addition, the prediction model of the service life was constructed for the proposed novel structural system with CFRP strip as an ICCP anode to provide reasonable suggestions for design.

## 2. Principle of ICCP with CFRP Strip as Anode

For the proposed ICCP-CS system in Figure 1, the positive and negative terminals of an external DC source were connected to the CFRP strip and the steel bar, respectively. A whole circuit can be formed with concrete (electrolyte), a CFRP strip (anode), and rebar (cathode). Thus, the rebar is forced to become the cathode with an impressed current, which makes the steel’s potential more negative to the corrosion-immune domain and inhibits further corrosion [27].

As depicted in Figure 1c, electrons flow from the steel bar to the steel–concrete interface, and the cathodic reaction (i.e., reduction reaction) is increased, generating hydroxide ions from oxygen and water, i.e.,

(1) $$O_{2}+2H_{2}O+4e^{-}\to 4{\mathrm{OH}}^{-}$$

(2) $$2H_{2}O+2e^{-}\to 2{\mathrm{OH}}^{-}+H_{2}$$

For the CFRP strip, the oxygen evolution reaction (i.e., oxidation reaction) is produced at the anode surface, generating oxygen and accompanied by chlorine when chloride ion exists, i.e., Equations (3) and (4). Therefore, a certain degree of acidification may occur in the CFRP–concrete interface strip anode and concrete, which may affect the corresponding bond behavior. In addition, hydroxide ions (i.e., OH^−^) migrated through the concrete to the anode and further oxidized to oxygen and electrons [28] (i.e., Equation (5)).

(3) $$2H_{2}O\to 4H^{+}+4e^{-}+O_{2}$$

(4) $$2{\mathrm{Cl}}^{-}\to {\mathrm{Cl}}_{2}+2e^{-}$$

(5) $$4{\mathrm{OH}}^{-}\to O_{2}+2H_{2}O+4e^{-}$$

The transfer of electric current in concrete is generated by ion migration in the pore solution, where the cation (e.g., Na^+^, K^+^) migrates to the rebar’s surface and the anion (e.g., OH^−^, Cl^−^) migrates in the opposite direction. Due to their high concentration and mobility, hydroxide ions (OH^−^) carry most of the circulating current. Electrons then flow to the DC power, closing the circuit. Due to the action of current circulation, the cathodic reaction of the steel bar occurs quickly while the anodic reaction (i.e., Equation (3)) is inhibited. Relatively moderate current density can restore passivation and inhibit the corrosion of steel bars. Other beneficial effects were also produced by cathodic protection. For example, ionic migration and cathodic reaction lead to the increase in hydroxide ion concentration and the decrease in chloride ion content in the cathode, resulting in an increase in the alkalinity (i.e., PH) of concrete around the steel bar.

(6) $$\mathrm{Fe}\to {\mathrm{Fe}}^{2+}+2e^{-}$$

## 3. Test Program

### 3.1. Specimen Preparation

**Figure 2:**
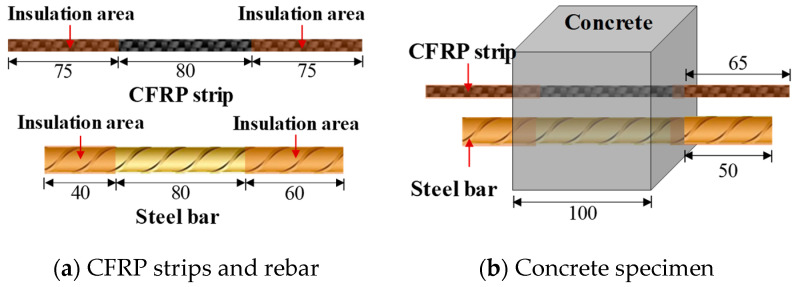
Specimen design diagram (unit: mm).

A total of 27 cube concrete blocks with a side length of 100 mm were cast, as presented in Figure 2. A CFRP strip, produced by Nanjing Haituo Composite Materials Company (Nanjing, China), with a length of 230 mm, a width of 15 mm, and a thickness of 0.168 mm was arranged on the inner side edge of the concrete block. A steel bar with a length of 180 mm and a diameter of 12 mm was set in the axial center of the concrete block. The middle region of the CFRP strip with a length of 80 mm belongs to the anodic polarization acceleration zone, while the non-test area with a length of 75 mm at both ends was covered with insulating tape and epoxy resin (see Figure 2a). To protect the rebar outside the concrete from corrosion, the corresponding parts were insulated in the same way. In this study, to identify the effect of different concrete environments on the performance of the CFRP strip anode, three types of concrete were investigated: traditional OPC concrete, engineered cementitious composite (ECC) concrete, and geopolymer concrete.

### 3.2. Material Properties

The mixture of different concretes is shown in Table 1. After curing for 28 days, the compressive strength of traditional OPC, ECC, and geopolymer concrete cubic blocks with a length of 100 mm was tested as 55.72 MPa, 61.28 MPa, and 43.5 MPa, respectively, according to GB/T 50081-2019 [29]. Based on standards GB/T 228-2010 [30] and GB/T 30022-2013 [31], the steel bars and CFRP strips were tested, with the corresponding mechanical properties shown in Table 2.

### 3.3. Accelerated Anodic Polarization Test

**Figure 3:**
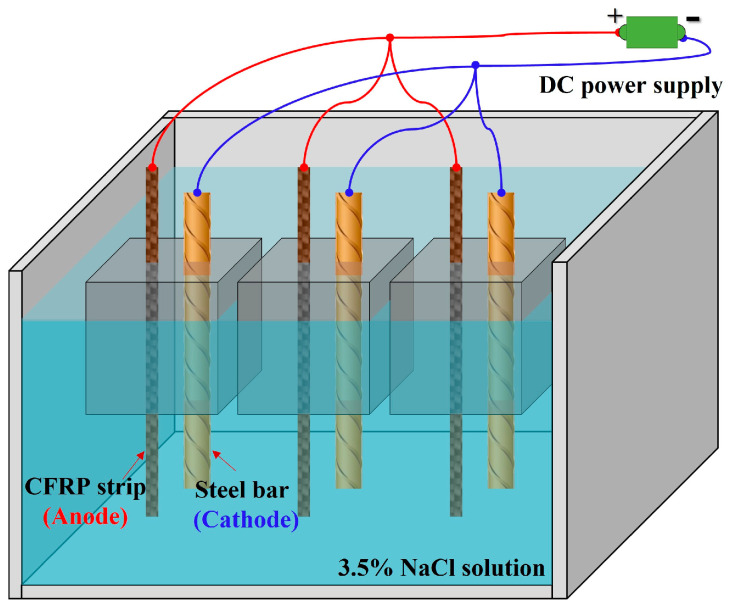
Anode polarization test.

As shown in Figure 3, the accelerated anodic polarization test with constant current was conducted for the concrete specimens after 28 days of curing. The test specimen was immersed in the 3.5% wt.% NaCl solution [7,22,32,33], which was used to simulate the corrosive marine environment. The CFRP strip and steel bar were connected to the positive and negative electrodes of the power supply to function as the anode and cathode in the ICCP system, respectively. Combined with the solution within the pores of the concrete, a whole circuit can be formed. The regions of steel bars and CFRP strips outside the concrete were covered with insulating tape and epoxy resin, as illustrated in Figure 2a. To identify the evolution of the properties for the CFRP strip anode in the proposed ICCP system within a reasonable test period, the maximum anode current density with a value of 108 mA/m^2^ specified in NACE SP0290-2000 [34] was magnified by 15, 30, 60, and 120 times, respectively. Thus, the applied anode current densities were 1620, 3240, 6480, and 12,960 mA/m^2^, with the corresponding output currents of the DC power supply being 2, 4, 8, and 16 mA, respectively. The detailed design is shown in Table 3. The letters C, E, and G represent the traditional OPC, ECC, and geopolymer concrete, respectively; the number (0, 15, 30,60, and 120) following the letter M indicates the amplification factor of the maximum anode current density, respectively; and three identical specimens were labeled a, b, and c. The test lasted for 30 days, and the feeding voltage was recorded every three days.

**Figure 4:**
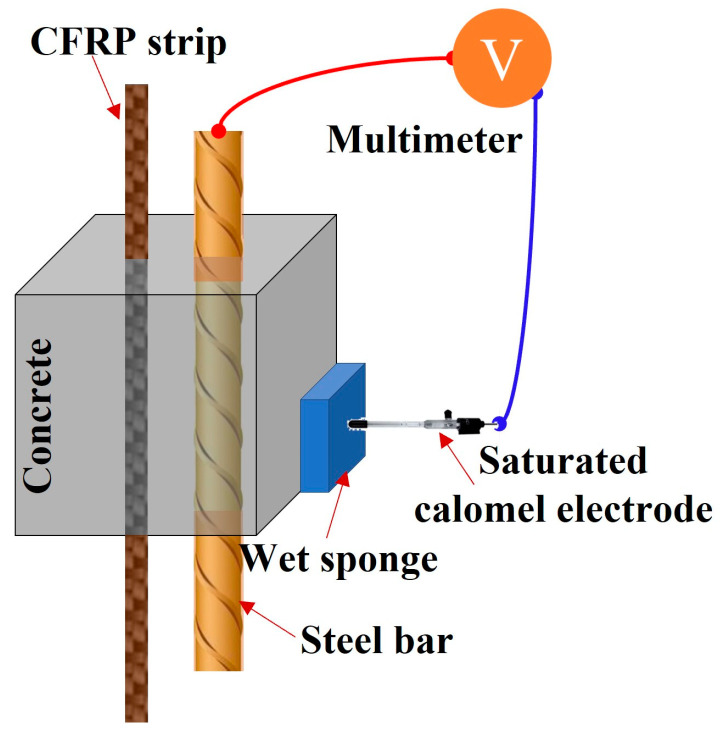
Electrochemical potential measurement.

To monitor the corrosion state of the steel bar, the corresponding electrochemical potentials (i.e., open circuit potential, instant-off potential, and depolarization potential) of the steel bar were measured every three days. The instant-off potential was recorded between 0.2 s and 1 s after switching off the DC power supply. The depolarization potential was recorded 4 h after the disconnection of the DC power supply to eliminate the effect of ICCP. As shown in Figure 4, the reference electrode is the saturated calomel electrode (SCE).

### 3.4. Electrochemical Impedance Spectroscopy

**Figure 5:**
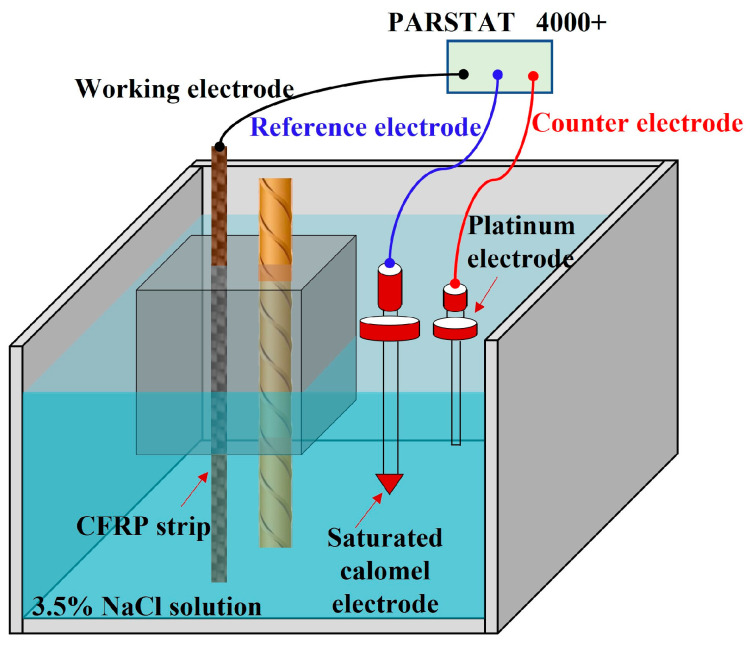
Schematic diagram of the electrochemical impedance spectroscopy test.

To study the influence of ICCP on the performance deterioration of the anode and system impedance of CFRP strips in a concrete environment, an electrochemical impedance spectroscopy (EIS) test was conducted before and after the accelerated anodic polarization test. As shown in Figure 5, the three-electrode system was used, with the CFRP strip, SCE, and platinum electrode acting as the working, reference, and counter electrodes, respectively. Before the EIS test, the DC supply was switched off for 4 h to allow the CFRP strip anode to tend to a steady state in this system. The scanning frequency of the EIS test ranges from 10^−2^ Hz to 10^4^ Hz, according to existing studies [11,35].

### 3.5. Uniaxial Tensile Test

**Figure 6:**
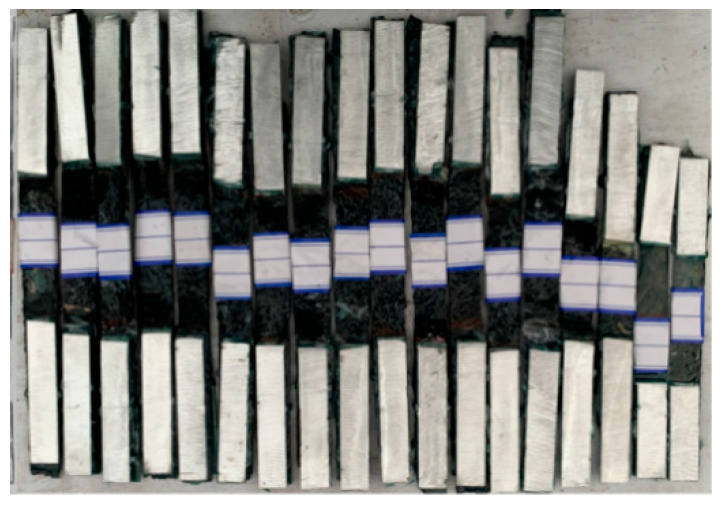
Uniaxial tensile specimens of CFRP strip anode.

To quantify the effect of anodic polarization on the mechanical properties of CFRP strips in concrete, the uniaxial tensile test was performed by removing the CFRP strip from the concrete block after the anodic polarization test. As shown in Figure 6, to avoid the crush at both ends of the CFRP strip, an aluminum sheet with a width of 15 mm and a length of 75 mm was adopted to clamp the end parts. The uniaxial tensile test of CFRP strips was conducted by an electro-hydraulic servostatic universal testing machine with a loading rate of 0.3 mm/min.

### 3.6. Acidification Test

In the ICCP process for RC structures, the anodes undergo oxidation (see Equations (3) and (4)), and the generated hypochlorous acids dissolve in the concrete pore solution, resulting in the reduction in concrete PH and the acidification of the anode interface. Therefore, the anode–concrete interface may be affected by the ICCP. In this study, a 0.5% phenolphthalein reagent was sprayed on the crushing section of concrete to assess the acidification of the anode–concrete interface after the anodic polarization of the CFRP strip.

## 4. Results and Discussion

### 4.1. Experimental Phenomena

#### 4.1.1. Anodic Polarization Phenomenon

**Figure 7:**
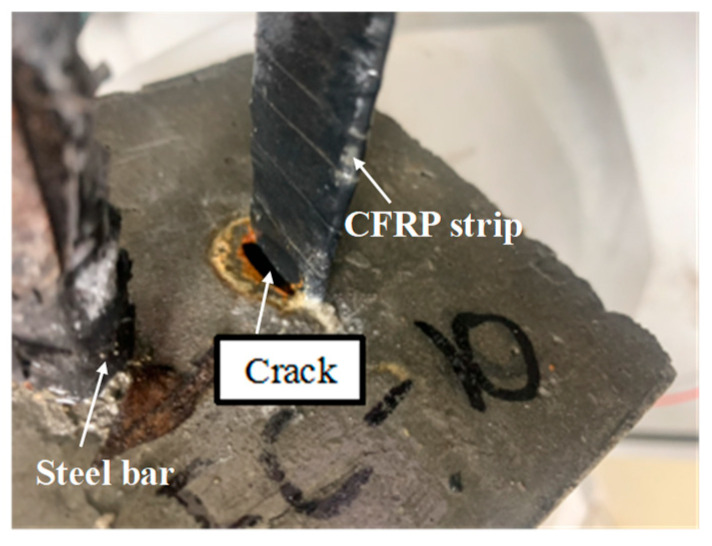
Specimens after the anode polarization test.

**Figure 8:**
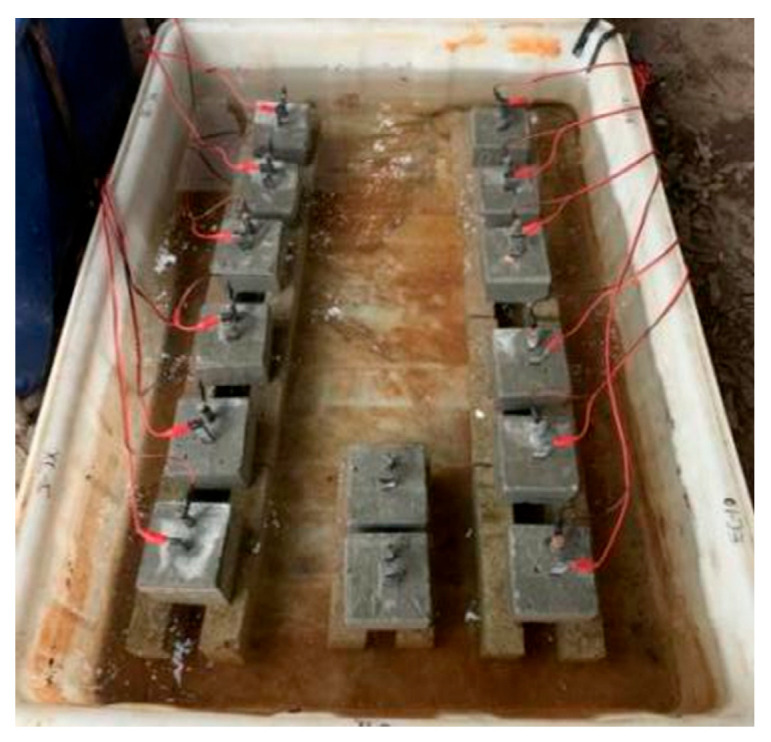
Electrochemical testing.

Figure 7 presents the phenomenon in the anodic polarization test. Many brown bubbles occurred on the surface between the CFRP strip anode and the concrete, followed by visible cracks. The connection point between the CFRP strip and the wire clamp head was seriously damaged, with loose fibers and yellow-brown rust. Some of the wire clamps for the G-M120 specimens were completely damaged, leading to large fluctuations in the feeding voltage. Compared with the anodic polarization in practical ICCP systems, the ion transportation, CFRP degradation, and gas escape have been accelerated in the anodic polarization with large current densities, leading to the underestimation of the long-term performance for CFRP strip anodes. This discrepancy should be considered in future studies. Electrochemical testing was conducted on the specimens following cathodic polarisation, as illustrated in Figure 8.

#### 4.1.2. Failure Mode

**Figure 9:**
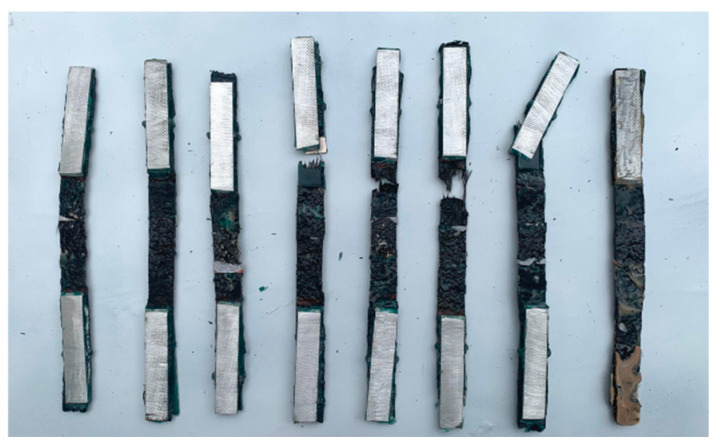
Failure mode of CFRP strip test specimens.

At the beginning of the uniaxial test for CFRP strips, the load increased linearly. As time went on, resin cracking appears, leading to small fluctuations occurring on the load curve. As the load continued to increase, part of the fiber bundles failed with an intense cracking sound, resulting in the fracture of all the CFRP strips. As shown in Figure 9, all the CFRP strips after anodic polarization failed in a brittle form with different fracture locations.

### 4.2. Feeding Voltage

**Figure 10:**
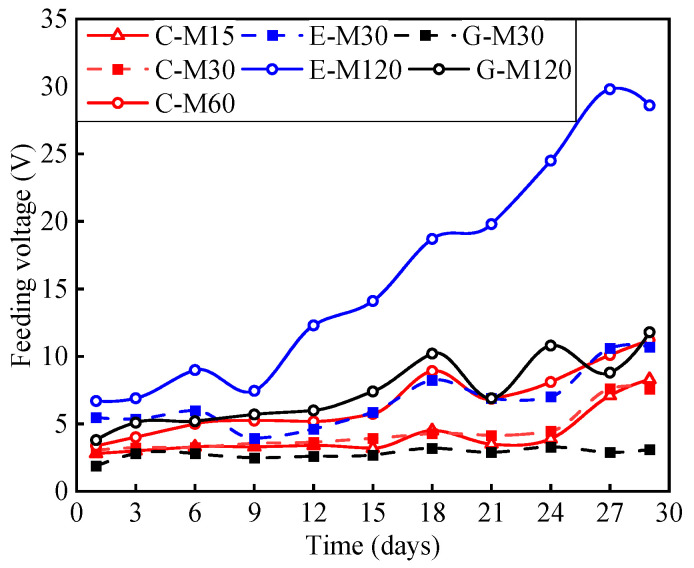
Variation in the feeding voltage.

Figure 10 shows the variation in the feeding voltage for each specimen during the test. The voltage is the value of the applied current multiplied by the electric resistance; thus, the variation in the feeding voltage is the variation in the system’s resistance with constant current in the anodic polarization test. The feeding voltage increased gradually with the increase in the applied current density. Furthermore, due to the excellent impermeability of ECC concrete and the low electrolyte content, the feeding voltage of the E-M specimen was larger than that of the C-M and G-M specimens under the same current density. Except for the specimen E-M120, the feeding voltage of the remaining specimens was relatively stable during the early stage while fluctuating obviously during the later stage.

For specimens with OPC concrete, the largest fluctuation of C-M15, C-M30, and C-M60 is 5.5 V, 4.56 V, and 7.83 V, respectively. For specimens with ECC concrete, the largest fluctuation of E-M30 and E-M120 was 6.77 V and 23.1 V, respectively. The large fluctuation of the feeding voltage for specimen E-M120 was due to the damage of the wire clamp with an excessive anode current. For specimens with geopolymer concrete, the largest fluctuation of G-M30 and G-M120 was 1.4 V and 8 V, respectively. As mentioned in [36], the feeding voltage will increase sharply to 3 V when the anode is seriously deteriorated. Thus, the CFRP strip anode suffers from serious deterioration when excessive currents are applied in the ICCP system. In general, as an auxiliary anode material, the CFRP strip shows satisfactory conductivity and relatively low output resistance.

### 4.3. Electrochemical Potentials

#### 4.3.1. Instant-Off Potential

**Figure 11:**
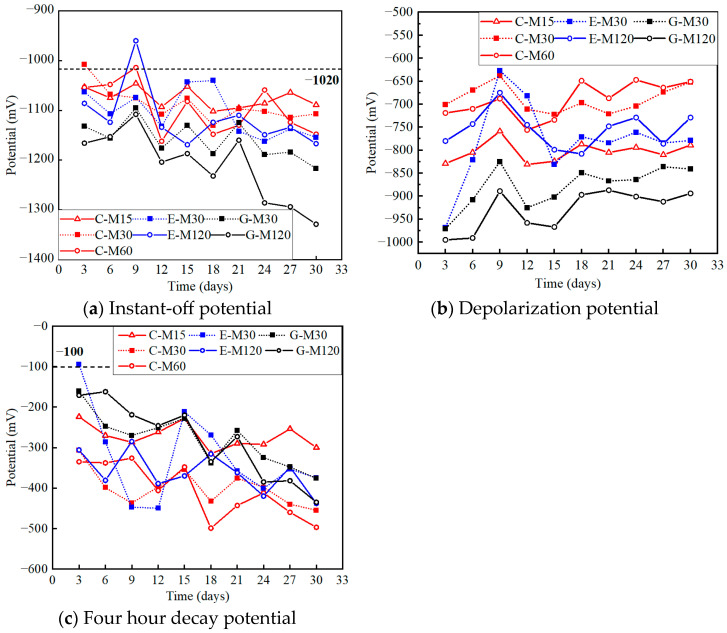
Variation in electrochemical potentials for each specimen.

Figure 11a illustrates the rebar’s instant-off potential (vs. SCE) during the anodic polarization test. Considering the variability across three identical specimens, the average values of the potential were selected in the following analysis. The ICCP effectiveness could be judged by the BS EN 12954 standard [37]: most effective if the rebar’s potential is located in the range of −670–−1020 mV (vs. SCE); not effective if the rebar’s potential is more positive than −670 mV (vs. SCE), while over-protection occurs for potentials more negative than −1020 mV (vs. SCE). All the instant-off potentials were more negative than −1020 mV, indicating that all specimens were in an overprotected state due to the excessive current densities applied in the accelerated anodic polarization test. Moreover, the potential developed in a negative direction as time increased, and the absolute value increased as the current density increased, as listed in Table 4.

#### 4.3.2. Depolarization Potential

Figure 11b plots the rebar’s depolarization potential (vs. SCE) during the anodic polarization test, with the detailed values listed in Table 4. As described in American standard ASTM C876-09 [38], the corrosion probability can be identified according to the rebar’s depolarization potential: 10% and 95% for potential more positive than −125 mV (vs. SCE) and negative than −275 mV (vs. SCE), respectively, but is uncertain for the potential between these two thresholds. The initial potentials of steel bars are more negative than −275 mV owing to the excessive current densities applied to the steel bars. However, the depolarization potential of the protected specimens gradually develops in a positive direction with time, which indicates that the corrosion is effectively suppressed, as demonstrated in the anodic polarization acceleration test. The actual corrosion state of the steel bar can be further identified qualitatively and quantitatively after the whole test, based on visual inspection, weighing, 3D scanning, and X-CT scanning.

#### 4.3.3. Four Hour Decay Potential

Figure 11 represents the 4 h decay potential during the 30-day test. The decay potential of steel bars is the discrepancy between the instant-off potential and the depolarization value, which is an important index for judging the effectiveness of ICCP. According to European standard EN 12696 [39], ICCP is effective when the corresponding 4 h decay potential is larger than 100 mV. As the ICCP test proceeded, the 4 h decay potential increased gradually (see Table 4), and was larger than 100 mV for all the protected specimens, verifying that the ICCP effectiveness in this system with CFRP strips as anode was obvious.

### 4.4. EIS Results

Electrochemical impedance spectroscopy tests were conducted for each specimen. Based on the real part (*Z’*) and imaginary part (*Z”*) of the impedance, the impedance modulus value (|*Z*|) and the corresponding phase angle (ϕ) can be assessed by:

(7) Z­2=Z′2+Z″2

(8) $$\phi =\frac{\arctan(\frac{-Z^{\prime \prime }}{Z^{\prime }})\times 180^{\circ }}{\pi }$$

**Figure 12:**
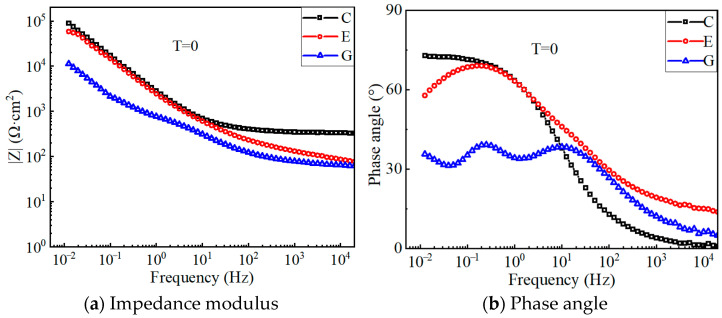
Bond plot for test specimens before the polarization test.

Figure 12 shows the Bode plots of the electrochemical impedance for each specimen before polarization. In general, an impedance phase angle close to 90° means that the electrode interface has a purely capacitive characteristic. The phase angles of each specimen are less than 90° (see Figure 12b), indicating that the CFRP strip anode deviates from the ideal capacitance [36]. This diffusion effect may be caused by the inhomogeneity of the solid electrode surface, the adsorption layer on the electrode surface, and the poor conductivity of the solution. The impedance modulus of the G-M specimens in the low-frequency range is approximately one order of magnitude smaller than that of the E-M and C-M specimens, which indicates that the equivalent electric double-layer capacitors of the CFRP solid electrode and electrolyte in the geopolymer concrete possess better charging and discharging capabilities.

**Figure 13:**
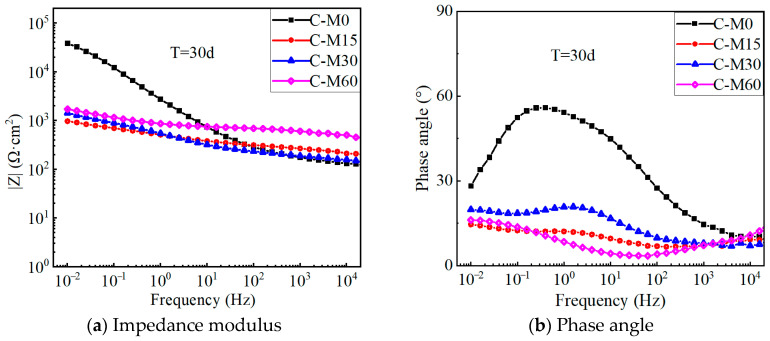
Bond plot of C-M specimen after 30 days of polarization.

Figure 13 shows the Bode plots of the C-M specimens after 30 days of polarization. In the low-frequency band, the impedance modulus of the C-M specimens decreases gradually with the increase in the applied polarization current density. For specimens treated with larger anode current densities, the electronic exchange at the CFRP–concrete interface is more thorough than that of the specimens with small anode current densities, which causes the decrease in the chloride ion concentration around the interface and the increase in the boundary resistance. In addition, the phase angle of the low-frequency band decreased from 65° to about 15°, since the polarization effect degrades the epoxy resin on the CFRP surface, increases the surface inhomogeneity, and causes a large variation in the surface properties of the CFRP electrode.

**Figure 14:**
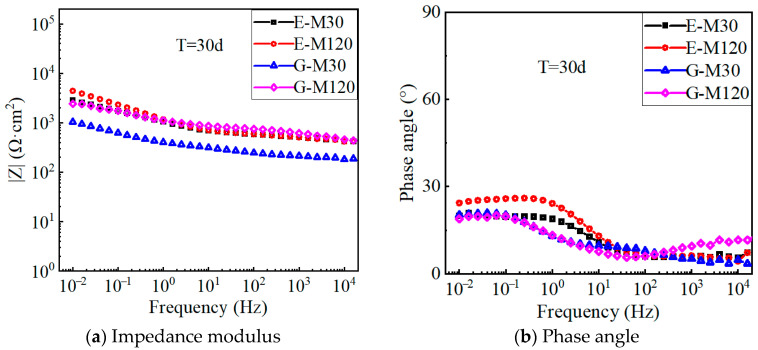
Bond plot of E-M, G-M specimen after 30 days of polarization.

Figure 14 shows the impedance modulus and phase angle for E-M and G-M specimens after polarization. The impedance modulus of the G-M specimen is smaller than that of the E-M specimen under equal polarization currents. This indicates that the equivalent double-layer capacitance of the CFRP solid electrode and the electrolyte in the geopolymer concrete has a better charging and discharging capability, leading to less system circuit resistance. In addition, the corrosion medium fully penetrates the solid electrode, coupled with the polarization effect, which results in the obvious reduction in the impedance modulus for each specimen. As the anode current density increases, the low-frequency impedance modulus increases gradually due to a more adequate electron exchange at the CFRP–concrete interface.

The impedance modulus is influenced by the coupling effect of the electrode polarization and the chloride concentration in the electrolyte. At the beginning of the polarization test, the carbon fiber in the CFRP electrode will not be consumed, and the degradation of the resin adhesive becomes a key factor affecting the impedance modulus. As polarization continues, more carbon fibers are exposed to participate in the electrode oxidation reaction, leading to a sudden decrease in the impedance modulus. When the polarization is sufficient, the impedance modulus is dominated by the chloride concentration. Thus, the chloride concentration around the electrode decreases as the polarization current increases, and the impedance modulus increases slightly. Furthermore, compared to the non-electrified specimen, the impedance modulus of the electrified specimen has a slight increase in the high-frequency range.

### 4.5. Uniaxial Tensile Test Results

**Figure 15:**
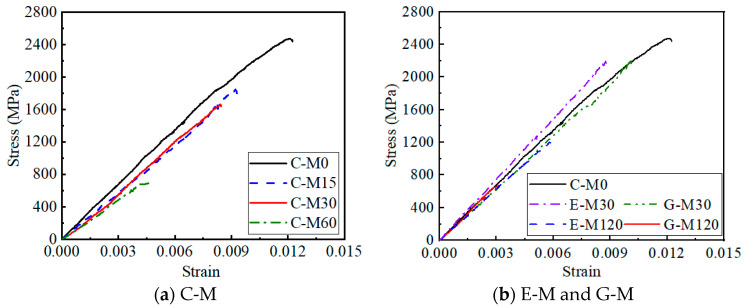
Stress–strain curve of CFRP strip anode.

Figure 15 shows the stress–strain curves of the CFRP strip anode after the anodic polarization test. The CFRP strip anode exhibits linear elastic characteristics during the uniaxial tensile test for each specimen. The ultimate strength of the CFRP strip anode after polarization gradually decreases with the increase in current density. This can be explained by the fact that the intense electrode reaction breaks the chemical bonding of the epoxy resin on the surface of the CFRP strip [16], making it a looser material. In addition, compared with traditional OPC concrete (i.e., C-M specimens), the ultimate strength of the CFRP strip shows a slight degradation for ECC and geopolymer concrete. For the geopolymer concrete with the anode current density amplified by 120 times, the CFRP strip shows a more severe degradation than ECC concrete under the same condition.

**Figure 16:**
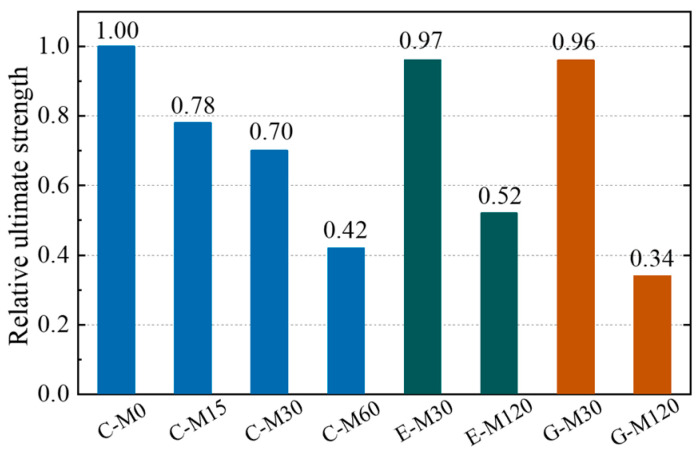
Relative ultimate tensile strength of CFRP strip anode.

Figure 16 presents the relative strength of the CFRP anode. Compared with the C-M0 specimen, the average ultimate strength of the CFRP strip for C-M15, C-M30, and C-M60 specimens decreased by 21.97%, 29.90%, and 57.80%, respectively. The average ultimate strength of the CFRP strip for E-M30 and GM30 specimens decreased by 3.18% and 3.61%, respectively. The average ultimate strength of the CFRP strip for E-M120 and G-M120 specimens decreased by 48.15% and 65.67%, respectively. Since the compactness of ECC and geopolymer concrete is superior to that of OPC concrete, and the anti-polarization ability of its internal CFRP strip is better than that OPC concrete; thus, the strength loss of E-M and G-M specimens is less than that of C-M under the same current density.

The long-term anodic polarization test is usually performed by magnifying the anode current density for a short duration [40]. Thus, there may be a difference between the accelerated deterioration and real deterioration for the anode material. To reveal the real deterioration of CFRP strip anode in practical engineering, normal current densities should be applied for a long-term duration, which should be investigated in future studies.

### 4.6. Acidification Analysis of CFRP–Concrete Interface

**Figure 17:**
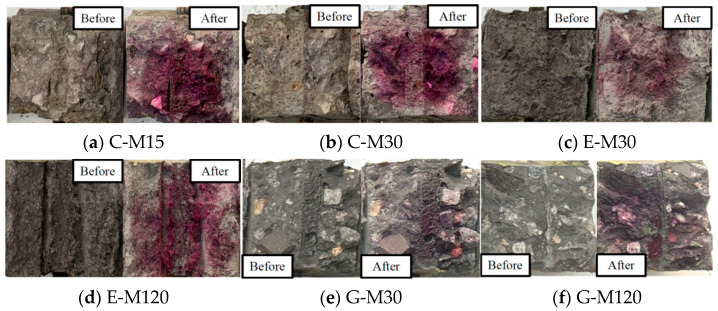
Interface acidification test results.

Figure 17 shows the results of acidification at the CFRP strip–concrete interface. Compared with the C-M15 specimen, the CFRP strip groove profile of the C-M30 specimen is clearly visible, indicating acidification occurs at the anode–concrete interface. As depicted in Figure 17c,e, the color distinction between the CFRP strip groove and the surrounding concrete is not obvious for E-M30 and G-M30 specimens, indicating that the acidification degree of the anode–concrete interface is weak. The CFRP strip groove profile of the E-M120 and G-M120 specimens is obvious, and the anode–concrete interface is seriously acidified (see Figure 17d,f). Under the same current density, the acidification degree of the interface between the CFRP strip anode and concrete in geopolymer and ECC concrete environments is higher than that of traditional OPC concrete. For the same type of concrete, the acidification degree of the CFRP–concrete interface deepens as the applied current density increases. To confirm the chemical degradation at the CFRP strip anode–concrete interface, quantitative measures (e.g., pH profiling, ion chromatography, and SEM/EDS mapping) accompanied by chemical analysis should be considered in future studies.

## 5. Numerical Analysis

### 5.1. Computational Model

**Figure 18:**
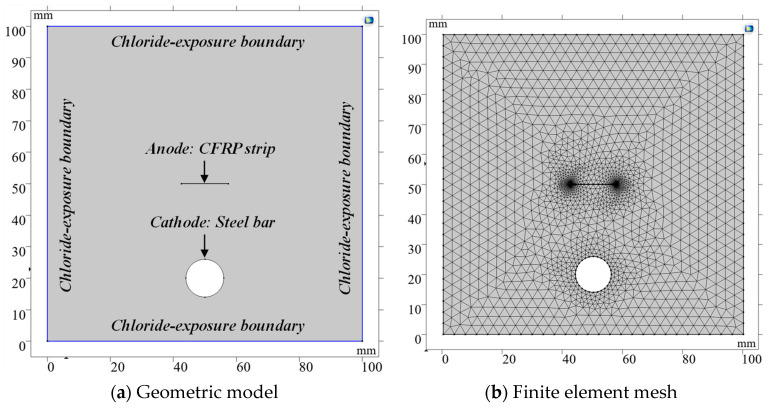
Geometric model and corresponding finite element mesh.

To further reveal the cathodic protection mechanism of CFRP strips as an anode, a numerical simulation analysis for the anodic polarization process was performed using COMSOL Multiphysics software in this section. A two-dimensional geometric model of the current-controlled ICCP, scaled to the test specimens, was established to improve convergence and reduce computational cost. The side and bottom faces of the RC blocks were designated as chloride-exposure boundaries, with concentrations for both Cl^−^ and Na^+^ fixed at 614 mol/m^3^ (3.5 wt% NaCl solution), as shown in Figure 18a. Free triangular mesh was employed to discretize the geometrical model, with refined mesh applied at the steel and CFRP surfaces, as illustrated in Figure 18b. The tertiary current distribution field was selected, as it can accurately describe the current and potential distribution in an electrochemical cell. This approach accounts for the individual transport of both charged species (ions) and uncharged species in the electrolyte, considering diffusion, migration, and convection through the application of the Nernst–Planck equations. The electrode kinetics for the charge transfer reactions were described using the predefined Tafel expressions. Three electrochemical reactions, at the CFRP–concrete interface, were considered: an oxygen evolution reaction (OER), chlorine evolution reaction (CER), and hypochlorous acid oxidation reaction (HCOR). At the steel–concrete interface, the reactions considered were iron dissolution, oxygen reduction reaction (OER), and hydrogen evolution reaction (HER).

**Figure 19:**
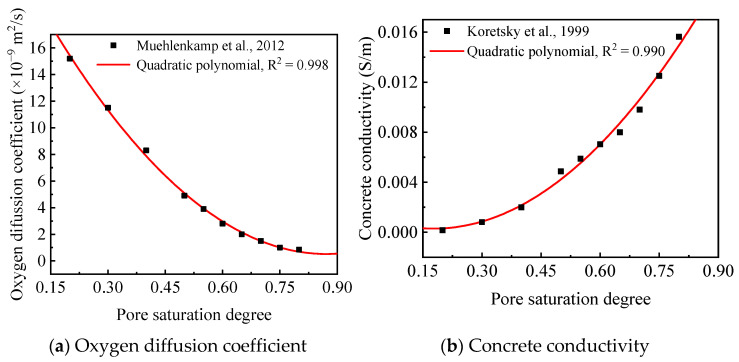
Oxygen diffusion coefficient and concrete conductivity in the numerical model [42,51].

The model inputs—diffusion coefficients *D_i_*, initial concentrations *c_ini_*_,*i*_, boundary concentrations *c*_0,*i*_ of each species, and the electrode kinetics parameters—are specified in Table 5 and Table 6, based on data from previous studies [40,41,42,43,44,45,46,47,48]. Concrete conductivity and oxygen diffusivity were treated as functions of pore saturation, as shown in Figure 19 [41,49,50]. The degree of saturation (S), an important parameter for characterizing the moisture in concrete, is influenced by the water-to-cement (w/c) ratio and concrete type. Notably, the presence of silica fume has been proven to cause a significantly higher saturation degree compared to ordinary concrete [51]. As indicated in Table 1, the water-to-cement (w/c) ratios for OPC and ECC concrete are 0.44 and 0.33, respectively. According to the relationship between the w/c ratio and S value reported in Ref. [51], the S values for OPC and ECC concrete were consequently set to 80% and 70%, respectively. For GPC concrete, the S value was assigned as 90% based on Ref. [52]. Corresponding oxygen diffusion coefficients were specified for each concrete type, as detailed in Table 5. The diffusion coefficients of Cl^−^ in the OPC concrete, the ECC concrete and the geopolymer concrete were taken as 1.20 × 10^−11^, 0.60 × 10^−11^, and 0.238 × 10^−11^ m^2^/s, respectively [40,43,46]. Diffusion coefficients of other ions, such as Na^+^, K^+^, Ca^2+^, OH^−^ and Fe^2+^, were determined based on Ref. [49], as listed in Table 6.

### 5.2. Effect of Current Density

Table 7 makes comparisons between the experimental and simulation results of specimens C-M15, C-M30 and C-M60 in terms of rebar potential and feeding voltage. The simulated rebar potential decreased from −1087.6 mV to −1182.9 mV as the impressed current increased from 2 mA to 8 mA. In the simulation, the rebar potential and external voltage remain constant, as material deterioration is not considered. The simulated rebar potential and feeding voltage closely approximate the experimental values, indicating that the COMSOL-based ICCP system is both reasonable and feasible.

**Figure 20:**
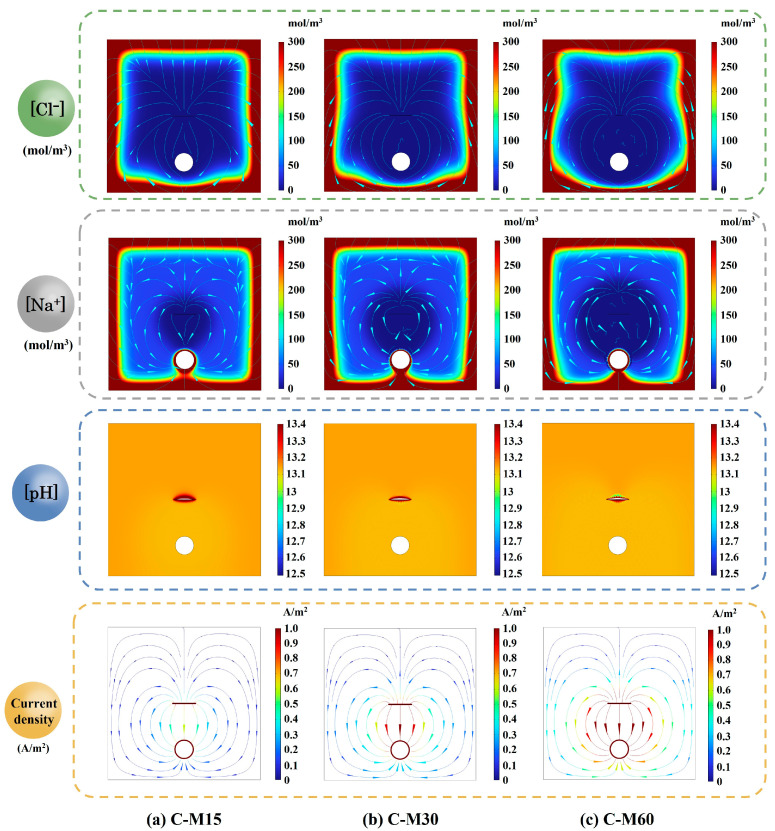
Distributions of Na^+^ and Cl^−^ concentrations, pH, and current density in OPC RC blocks after 30 days of anodic polarization.

Figure 20 compares the simulated spatial distributions of Na^+^ and Cl^−^ concentrations, pH, and current density in the OPC-RC blocks after 30 days of constant-current ICCP at imposed currents of 2, 4, and 8 mA (specimens C-M15, C-M30 and C-M60). The Cl^−^ and Na^+^ concentration patterns are governed by diffusion from the chloride-exposed surface and field-assisted ionic migration. The concentrations of Na^+^ and Cl^−^ are similar among the RC blocks across current levels, with higher concentrations near the chloride-exposure face and a significant drop as the distance from the chloride-exposure boundary increases, which aligns with the findings reported by Guo et al. [49].

The pH distribution plays a crucial role in electrochemical processes, particularly in understanding the acidification at the anode–concrete interface. At the steel surface, ORR tends to increase local pH, while the imposed electric field simultaneously drives OH^−^ to-ward the anode. As the applied current increases, the pH values remain stable around 13.1, indicating that the migration of OH^−^ is balanced with local generation. At the CFRP anode, a localized pH decrease is observed around the strip, indicating acidification at the CFRP–concrete interface. As the current increases, the acidification at the CFRP strip is more pronounced. This observation aligns with experimental results, thereby validating the accuracy and reliability of the simulation. Meanwhile, the adverse effects have been confirmed by some experimental results [49,53,54,55].

Figure 20 also illustrates that the current flows from the CFRP strip to the steel bar. Increasing the imposed current amplifies the potential gradient and concentrates current beneath the CFRP strip. The current density beneath the steel surface is significantly lower than the opposite surface, indicating that the outside surface of the steel bar, facing the concrete cover, experiences more severe corrosion compared to the opposite surface. Thus, the outside surface requires additional protective current [49].

### 5.3. Effect of Concrete Type

**Figure 21:**
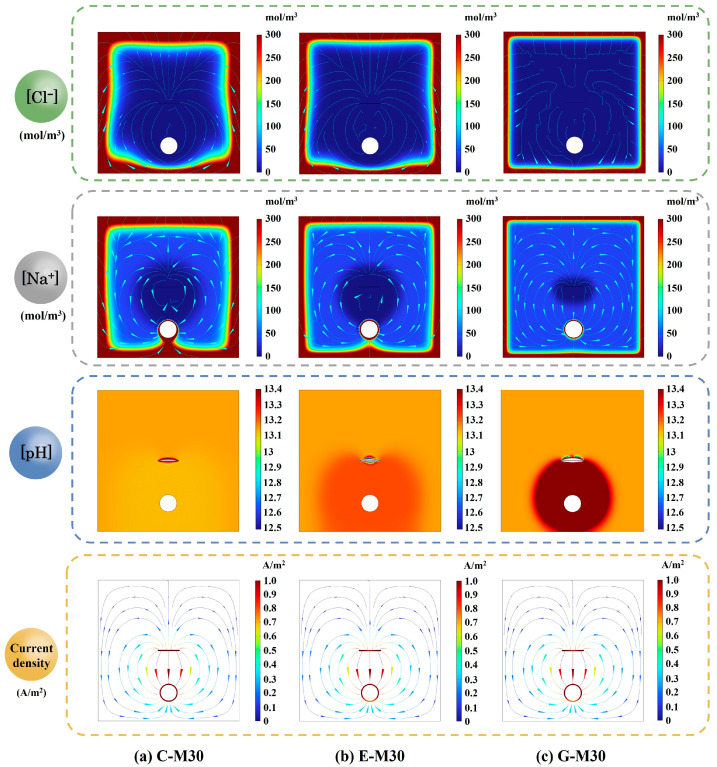
Distributions of Na^+^ and Cl^−^ concentrations, pH, and current density in different concretes after 30 days of anodic polarization.

Table 7 makes comparisons between experimental and simulation results for specimens C-M30, E-M30, and G-M60 in terms of rebar potential and feeding voltage. Overall, the simulated values obtained using COMSOL demonstrate a strong agreement with the experimental results. Similarly, the spatial distributions of Cl^−^ and Na^+^ concentrations, pH, and current density were simulated for different concrete (i.e., C-M30, E-M30, and G-M30), as shown in Figure 21. In all specimens, the highest concentrations appear near the exposure surface, with a gradual decline in depth. Relative to OPC concrete, both ECC and geopolymer concrete exhibit reduced Cl^−^ penetration toward the rebar, with the geopolymer exhibiting the lowest Cl^−^ concentration level in the vicinity of the rebar owing to its refined pore structure and higher resistivity. As expected for a cathodically polarized steel bar, Na^+^ migrates toward the steel bar, forming a cathodic accumulation zone. At the cathode, ORR generates OH^−^, while the imposed electric field drives OH^−^ toward the anode, resulting in a pH increase around the cathode. The smallest pH decrease occurs in C-M30 due to its higher ionic diffusion coefficients. Localized acidification is also observed around the CFRP strip. This acidified zone is more pronounced in E-M30 and G-M30, consistent with the experimental observations. In addition, the discrepancy in current density among the specimens is not significant.

## 6. Life Assessment of the Proposed ICCP System

### 6.1. Strength Prediction

Based on the results of the uniaxial tensile test obtained in Section 4.5, the ultimate strength for the CFRP strip anode under anodic polarization can be predicted as a function of the charge density *q*, i.e.,

(9) $$f_{C-u}/f_{RF-u}=1-0.34799q$$

(10) $$f_{E-u}/f_{RF-u}=1-0.13321q$$

(11) $$f_{G-u}/f_{RF-u}=1-0.1812q$$

in which *f_RF_*_-*u*_ denotes the ultimate strength of the CFRP strip in traditional OPC concrete without anodic polarization, *f_C_*_-*u*_, *f_E_*_-*u*_, and *f_G_*_-*u*_ represent the ultimate strength of the CFRP strip after anodic polarization in traditional OPC concrete, ECC concrete, and geopolymer concrete, respectively.

**Figure 22:**
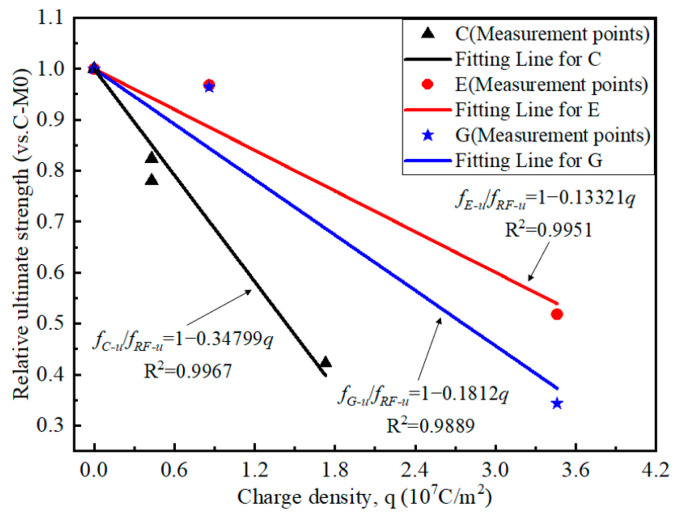
Prediction of the ultimate strength for the CFRP strip anode.

An obvious discrepancy exists in the deterioration trend of the ultimate strength for the CFRP strip anode in different concrete environments, as illustrated in Figure 22. For example, when the total charge density is 1.73 × 107 C/m^2^, the ultimate strength of CFRP strips is 40%, 77%, and 69% of the initial value for C-M, E-M, and G-M specimens, respectively. When the charge density is 3.46 × 107 C/m^2^, the relative ultimate strength of the CFRP strips is 54% and 37% for the E-M and G-M specimens, respectively.

For fixed anodic polarization, the residual ultimate strength of the CFRP anode is highest in the ECC concrete environment, followed by the geopolymer concrete and traditional OPC concrete. Moreover, the deterioration rate of the CFRP strip anode in ECC concrete is lower than that in geopolymer concrete and traditional OPC concrete. For a fixed relative residual strength, the flux density of the anode in ECC concrete is significantly larger than that in geopolymer concrete and traditional OPC concrete, which indicates that the ICCP system with CFRP strips as anodes has a longer service life in ECC concrete.

### 6.2. Service Life Assessment

#### 6.2.1. Prediction Model

It is noted that the service life of the ICCP system could be estimated based on the electric flux transferred between the anode and the electrolyte interface [56]. In the ICCP process, the reaction between the cathode and anode is balanced with an equal charge quantity. Therefore, the electric flux *Q_anode_* passing through the anode’s surface is equal to the electric flux *Q_cathode_* transferred by the cathode, i.e.,

(12) $$Q_{anode}=Q_{cathode}$$

with the electric flux of anode *Q_anode_* and cathode *Q_cathode_* expressed by:

(13) $$Q_{anode}=q_{anode}\times A_{a}$$

(14) $$Q_{cathode}=n\times i_{p}\times t_{life}\times A_{steel}$$

in which, *q_anode_* is the anode flux density; *A_a_* is the effective working area of the CFRP strip anode; *n* is the number of steel bars; *A_steel_* is the surface area of a single steel bar; *i_p_* is the protection current density; *t_life_* is the predicted lifetime of the proposed ICCP system.

**Figure 23:**
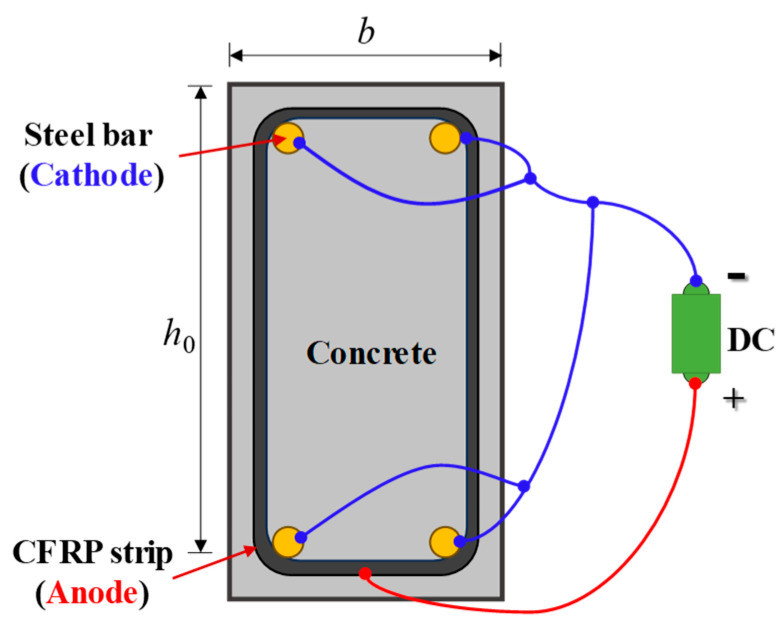
Construction model for evaluating service life (unit: mm).

To investigate the predicted lifetime of the proposed system, and to provide reasonable suggestions for the design in practical engineering, an RC beam with a height, width, and cover thickness of 250 mm, 120 mm, and 20 mm, respectively, is taken as an example in the following analysis. As illustrated in Figure 23, four rebars with a diameter of d were used as cathodes for the ICCP system, and the CFRP strips with an interval of s as the anodes of the ICCP system. The CFRP strip is impregnated with conductive resin adhesive and laminated in four layers to form a sleeve.

It is noted that, the total electricity of the steel bars and the CFRP anode in the ICCP process are related to the reinforcement ratio and the stirrup ratio, respectively. The surface area of a single steel bar *A_steel_* and the effective working area of the CFRP strip anode *A_a_* can be expressed as Equations (15) and (16), respectively.

(15) $$A_{steel}=\sqrt{\frac{4\pi A_{c}\rho }{n}}$$

(16) $$A_{a}=\frac{A_{a1}}{A_{sv}}\rho_{sv}b$$

where *A_c_* and *b* denote the area and the width of the cross-section of the RC beam, respectively; *ρ* and *ρ_sv_* denote the reinforcement ratio and stirrup ratio of the RC beam, respectively; *A_sv_* is the total cross-sectional area of each limb of stirrups arranged in the same cross-section; and *A_a_*_1_ is the anode working area of a single CFRP strip stirrup.

Therefore, combining Equations (13)–(16), the predicted service life of the proposed ICCP system with CFRP strip as anode can be expressed as:

(17) $$t_{life}=\frac{q_{anode}}{i_{p}}\times \frac{1}{\sqrt{4\pi nA_{c}\rho }}\times \frac{A_{a1}b}{A_{sv}}\rho_{sv}$$

#### 6.2.2. Parameter Analysis

To maintain the structural reinforcement function of the CFRP strip, an assumption was adopted here: Once the ultimate strength of the CFRP strip decreases by 40%, then the service life of the ICCP system concludes. As can be seen in Figure 22, for a relative residual strength of 60%, the corresponding system anode current density qanode in traditional OPC concrete, ECC concrete, and geopolymer concrete environments are 1.149 × 10^7^ C/m^2^, 3.003 × 10^7^ C/m^2^, and 2.208 × 10^7^ C/m^2^, respectively. To give reasonable suggestions for practical engineering, an ICCP current density ip of 20 mA/m^2^ was selected, which is a value used in practical engineering for ICCP [57]. Moreover, the ranges of the reinforcement ratio and stirrup ratio were selected as 0.3–3% and 1.2–4.8%, respectively, to provide suitable suggestions for the design of RC structures.

**Figure 24:**
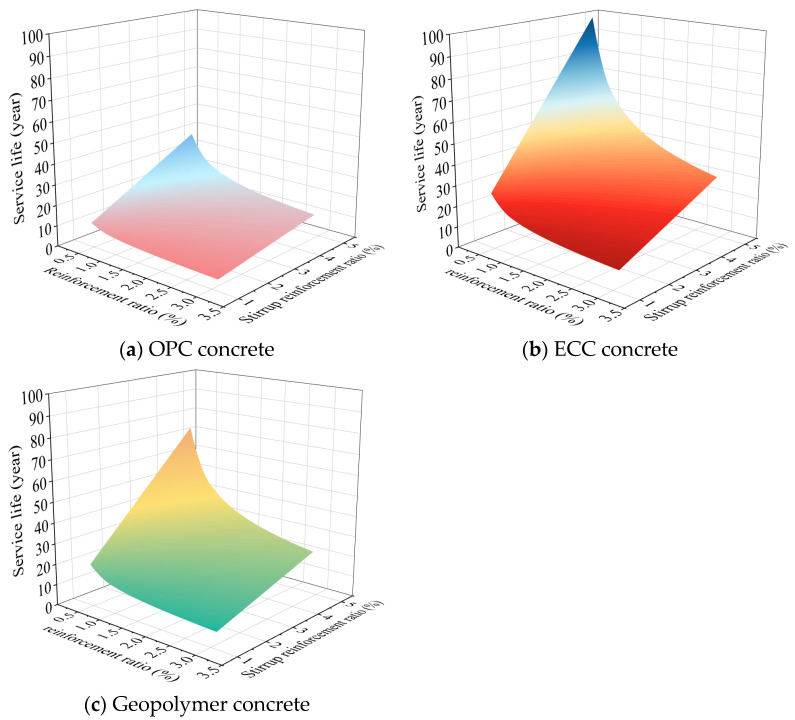
Response surface of the predicted lifetime for the ICCP system in different concrete.

Figure 24 presents the response surface of the predicted service life for the proposed ICCP system in different concretes. Under the same conditions, the predicted system’s service life is the longest for ECC concrete, followed by geopolymer concrete, and finally OPC concrete. The predicted service life of the ICCP system decreases nonlinearly as the reinforcement ratio increases, while it increases linearly as the stirrup ratio increases. Thus, the ratio of the anode area (i.e., CFRP strip stirrup) to the cathode area (i.e., steel bar) shows a positive effect on the predicted service life of the proposed ICCP system.

**Figure 25:**
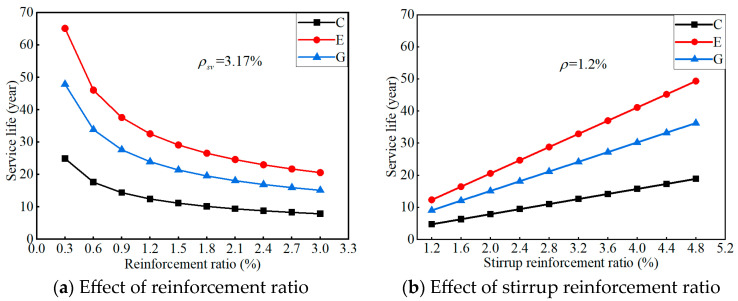
Effect of reinforcement ratio and stirrup ratio on system’s service life.

To quantify the effect of the reinforcement ratio and stirrup ratio on the predicted lifetime for the ICCP system, a specific case of the RC beam was considered with a stirrup spacing s of 100 mm (i.e., the stirrup ratio ρsv = 3.17%) and a rebar diameter d of 12 mm (i.e., the reinforcement ratio ρ = 1.2%). The results are shown in Figure 25. For this case, the predicted service life of the system is 12.5 years, 24 years, and 32.6 years for traditional OPC concrete, geopolymer concrete, and ECC concrete, respectively. Compared with traditional OPC concrete, the predicted service life increased by 92% and 161% for geopolymer concrete and ECC concrete, respectively, indicating the promising application potential of a CFRP strip anode in RC structures. Furthermore, the discrepancy of the predicted service life in different concrete environments decreases gradually as the reinforcement ratio increases, while it increases gradually as the stirrup ratio increases. Therefore, the reasonable selection of reinforcement ratio and CFRP stirrup ratio is the key to ensuring the safe service of the RC structure with CFRP strips as an anode, by considering the dual functions of the CFRP material.

**Figure 26:**
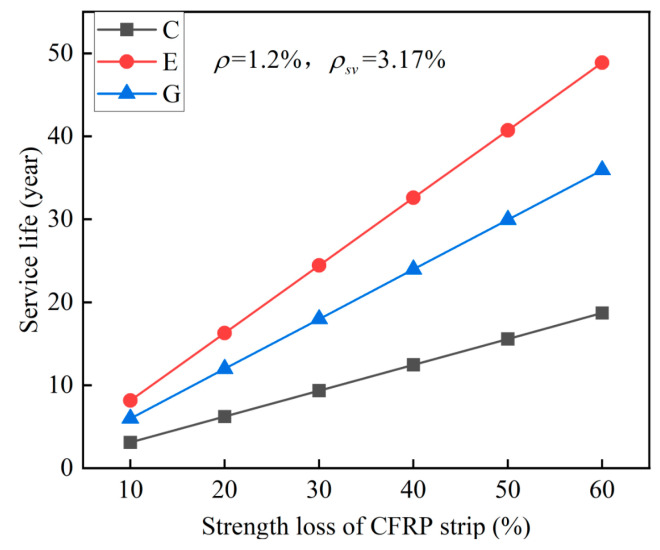
Effect of the strength loss of CFRP strip on system’s service life.

The failure criterion (i.e., the threshold of strength loss of CFRP) for the proposed system should be determined based on the material deterioration and structural bearing capacity simultaneously. Thus, to quantify the effect of the strength loss of the CFRP strip anode on service life, a sensitive analysis was performed here with the result shown in Figure 26.

## 7. Conclusions

Based on the exceptional electrical conductivity and mechanical properties of CFRP materials, a novel structural-functional integrated CFRP RC structure was proposed in this study. In this structural system, CFRP strips are used as both concrete stirrups and an auxiliary anode, ensuring the service safety of the structure and enhancing the application value of CFRP materials in civil engineering. The electrochemical and mechanical properties of the CFRP strip anode were investigated experimentally and analytically herein. Several conclusions can be obtained:

(1) As anodes in ICCP systems, CFRP strips exhibit excellent conductivity and relatively low output resistance within concrete environments. Notably, ECC concrete exhibits higher feeding voltages due to its superior impermeability and lower electrolyte content. Furthermore, the significant ICCP effectiveness was verified by the electrochemical potential.
(2) EIS results indicate that enlarged anode current densities enhance electron exchange at the CFRP/concrete interface, leading to a gradual increase in the low-frequency impedance modulus. The impedance modulus of geopolymer concrete is lower than that of ECC concrete for the same current density.
(3) Due to the deterioration of CFRP strips during the accelerated anodic polarization, the strength of the CFRP strips decreases gradually as the current density increases. For the specimens with a current density enlarged by 30 times, the strength of the CFRP strip in geopolymer and ECC concrete is about 38% higher than that of ordinary concrete. Moreover, the deterioration rate of the CFRP strip in ECC concrete is lower than that of geopolymer concrete.
(4) The predicted service life of the proposed ICCP system decreases as the reinforcement ratio increases, while it increases as the stirrup ratio increases. The proposed ICCP system has the longest service life in ECC concrete, followed by geopolymer concrete and OPC concrete: Compared with OPC concrete, the predicted service life for the specific case increased by 92% and 161% for geopolymer concrete and ECC concrete, respectively.

However, several limitations exist in this study because of simplifications and assumptions. To reflect the real deterioration of a CFRP strip anode in practical engineering, anodic polarization should be performed with normal current densities for a long-term duration, accompanied by a scanning electron microscopy test.

## Figures and Tables

**Table 1:** Mixture of different concrete (unit: kg/m^3^).

**(a) Traditional OPC Concrete**							
**Mixing Ratio**							**Compressive Strength**
**Cement**	**Sand**		**Aggregate**		**Water**		
489	764		934		215		55.72 MPa
**(b) ECC Concrete**							
**Mixing Ratio**							**Compressive Strength**
**Cement**	**Mineral Powder**	**Silica Fume**	**Cenosphere**	**Water**	**PE Fiber**	**HRWRA**	
700	188	230	216	230	20	45	61.28 MPa
**(c) Geopolymer Concrete**							
**Mixing Ratio**							**Compressive Strength**
**Aggregate**	**Sand**	**Fly Ash**	**Mineral Powder**	**NaOH**	**Sodium** **Silicate**	**Water**	
1120	530	369	92	14	110	105	43.5 MPa

**Table 2:** Mechanical properties of steel bar and CFRP strip.

Reinforcement	Diameter (mm)	Yield Strength (MPa)	Ultimate Strength (MPa)	Modulus (GPa)
Steel bar	12	416.54	578.75	201.51
CFRP strip	-	-	2491.92	227.02

**Table 3:** Experimental design of the anode polarization.

Specimen	Current (mA)	Current Density (mA/m^2^)	Electric Flux Density (10^7^ C/m^2^)	Duration (Day)
C-M0-a,b,c	0	0	0	-
C-M15-a,b,c	2	1620	0.43	30
C-M30-a,b,c	4	3240	0.86	30
C-M60-a,b,c	8	6480	1.73	30
E-M30-a,b,c	4	3240	0.86	30
E-M120-a,b,c	16	12,960	3.46	30
G-M30-a,b,c	4	3240	0.86	30
G-M120-a,b,c	16	12,960	3.46	30

**Table 4:** Electrochemical potential for each specimen (unit: mV).

Specimen	Instant-Off Potential		Depolarization Potential		Decay Potential	
	3 d	30 d	3 d	30 d	3 d	30 d
C-M15	−1053	−1089	−829	−789	−224	−300
C-M30	−1008	−1107	−701	−652	−307	−455
C-M60	−1054	−1148	−719	−651	−335	−497
E-M30	−1063	−1154	−968	−779	−95	−375
E-M120	−1086	−1167	−780	−729	−306	−438
G-M30	−1132	−1217	−971	−841	−161	−376
G-M120	−1166	−1329	−995	−894	−171	−435

**Table 5:** Diffusion coefficients and initial concentrations of the species in the present model.

Parameters	OH	Cl	Fe^2+^	Na^+^	Ca^2+^	O_2_	Ref.
Initial concentration *c_ini_*_,*i*_ (mol/m^3^)	140	0	0	40	50	0.156	[40]
Boundary concentration *c*_0,*i*_ (mol/m^3^)	0	614	0	614	0	0.268	
Diffusion coefficient *D_i_* in free water (×10^−9^ m^2^/s)	5.27	2.03	0.719	1.33	0.793	-	[42,44]
Diffusion coefficient *D_i_* in OPC concrete (×10^−11^ m^2^/s)	3.12	1.20	0.425	0.786	0.469	69	[40]
Diffusion coefficient *D_i_* in ECC concrete (×10^−11^ m^2^/s)	1.56	0.60	0.213	0.393	0.234	149	[46]
Diffusion coefficient *D_i_* in geopolymer concrete (×10^−11^ m^2^/s)	0.618	0.238	0.084	0.156	0.093	54	[43]

**Table 6:** Electrode kinetics parameters of the numerical model.

Reaction @Electrode	Equilibrium Potential *E_eq_* (SCE)	Tafel Slope (V/dec)	Exchange Current Density (A/m^2^)	Ref.
Fe → Fe^2+^ @Steel	−0.68	0.410	7.10 × 10^−5^	[41]
ORR @Steel	0.16	−0.180	7.70 × 10^−7^	[41]
HER @Steel	−0.24	−0.150	1.10 × 10^−2^	[41]
OER @CFRP	0.16	0.184	2.00 × 10^−4^	[47]
CER @CFRP	1.12	0.098	1.40 × 10^−2^	[45]
HCOR @CFRP	1.25	0.180	6.00 × 10^−6^	[48]

**Table 7:** Comparison between experimental and numerical results.

Specimen ID	Experimental Results		Numerical Results	
	Rebar Potential (mV_SCE_)	Feeding Voltage (V)	Rebar Potential (mV_SCE_)	Feeding Voltage (V)
C-M15	−1046–−1102	2.80–8.30	−1087.6	2.98
C-M30	−1008–−1130	3.05–7.61	−1134.3	4.11
C-M60	−1014–−1162	3.37–11.20	−1182.9	6.25
E-M30	−1063–−1162	3.05–7.61	−1136.9	5.32
G-M30	−1095–−1217	5.48–10.7	−1184.2	3.71

## Data Availability

The original contributions presented in this study are included in the article. Further inquiries can be directed to the corresponding author.
